# Rare Influenza A (H3N2) Variants with Reduced Sensitivity to Antiviral Drugs

**DOI:** 10.3201/eid1603.091321

**Published:** 2010-03

**Authors:** Clyde Dapat, Yasushi Suzuki, Reiko Saito, Yadanar Kyaw, Yi Yi Myint, Nay Lin, Htun Naing Oo, Khin Yi Oo, Ne Win, Makoto Naito, Go Hasegawa, Isolde C. Dapat, Hassan Zaraket, Tatiana Baranovich, Makoto Nishikawa, Takehiko Saito, Hiroshi Suzuki

**Affiliations:** Niigata University, Niigata, Japan (C. Dapat, Y. Suzuki, R. Saito, M. Naito, G. Hasegawa, I.C. Dapat, H. Zaraket, T. Baranovich, H. Suzuki); National Institute of Animal Health, Tsukuba City, Japan (T. Saito); Niigata Prefectural Institute of Public Health and Environmental Sciences, Niigata (M. Nishikawa); Sanpya Hospital, Yangon, Myanmar (Y. Kyaw); National Health Laboratory, Yangon (K.Y. Oo, N. Win); Central Myanmar Department of Medical Research, Nay Pyi Taw, Myanmar (Y.Y. Myint, N. Lin, H.N. Oo); 1 These authors contributed equally to this article.

**Keywords:** Influenza A virus, H3N2 subtype, zanamivir, amantadine, antiviral drug resistance, viruses, Myanmar, expedited, dispatch

## Abstract

In 2007 and 2008 in Myanmar, we detected influenza viruses A (H3N2) that exhibited reduced sensitivity to both zanamivir and amantadine. These rare and naturally occurring viruses harbored a novel Q136K mutation in neuraminidase and S31N mutation in M2.

Adamantanes and neuraminidase inhibitors (NAIs) are the 2 classes of drugs indicated for preventing or treating influenza virus infection. In 2005, the high prevalence of influenza viruses A (H3N2) with S31N mutation in M2 limited the effectiveness of amantadine (*1*,*2*). In 2008, the emergence of subtype H1N1 with H274Y mutation in neuraminidase (NA) raised concerns about the use of oseltamivir (*3*,*4*). On the other hand, the incidence of zanamivir-resistant viruses was low (*5*). In 1998, 1 case of zanamivir-resistant influenza B virus, which was isolated from an immunocompromised child who underwent prolonged zanamivir treatment, was reported (*6*). In 2008, subtype H3N2 with D151A/V mutations in NA demonstrated reduced zanamivir sensitivity by chemiluminescent NAI assay (*5*). Recently, zanamivir-resistant subtype H1N1 isolates with a novel Q136K mutation in NA were isolated in Oceania and Southeast Asia (*7*).

We report the detection of influenza viruses A (H3N2) harboring a Q136K mutation in NA and an S31N mutation in M2, which respectively confer reductions in zanamivir and amantadine susceptibility. In 2007 and 2008, we performed phenotypic and genotypic analyses in characterizing these viruses from Myanmar.

## The Study

Nasopharyngeal swabs were collected from patients with influenza-like illness at Sanpya Hospital in Yangon, Myanmar, and outpatient clinics affiliated with the Department of Medical Research (Central Myanmar) in Nay Pyi Taw. Rapid test kit–positive samples were sent to Niigata University, Japan, for subsequent analyses. Virus isolation and subtyping PCR were performed as previously described (*8*). The NAI susceptibility test was performed by a fluorescence-based NA activity assay that measures the 50% inhibitory concentration (IC_50_) by using zanamivir and oseltamivir carboxylate (*9*). All samples were assayed in duplicates in >2 independent experiments. A sample was considered an extreme outlier if its IC_50_ value was 10× higher than the mean values for sensitive strains with >3 interquartile range from the 25th and 75th percentiles in the box-and-whisker plot analysis (*9*). So far, all known NAI-resistant viruses are extreme outliers (*10*). Screening for S31N mutation in M2 was done by cycling probe real-time PCR (*11*). Sequencing and phylogenetic analysis of the hemagglutinin (HA) and NA genes were performed as previously described (*8*).

A total of 253 and 802 rapid test kit–positive samples were collected in Myanmar in 2007 and 2008, respectively. Of these, 64 isolates of subtype H3N2 were detected in 2007 and 211 in 2008. NAI susceptibility assay showed 1 (1.5%) isolate (A/Myanmar/M187/2007) with a zanamivir IC_50_ value of 59.72 nM, which was collected in August 2007, and 1 (0.5%) isolate (A/Myanmar/M114/2008) with a zanamivir IC_50_ of 33.37 nM, which was collected in July 2008. These isolates respectively demonstrated a 53× and 30× reduction in zanamivir susceptibility (Table) and were extreme outliers (data not shown). On the basis of cycling probe real-time PCR assay, these viruses had an S31N mutation in M2, which confers resistance to amantadine. All subtype H3N2 viruses analyzed in this study remain sensitive to oseltamivir carboxylate (Table).

**Table:** Characteristics of subtype H3N2 influenza viruses with Q136K mutation in NA and S31N substitution in M2*

Strains	Passage history	NA mutation	IC_50_s of NA inhibitors				Amantadine sensitivity† (M2 mutation)
			Zanamivir, nM ± SD	Fold change	Oseltamivir, nM ± SD	Fold change	
All NAI-sensitive subtype H3N2 isolates‡	MDCK2	None	1.12 ± 0.40	1	0.86 ± 0.44	1	Resistant (S31N)
A/Myanmar/M187/2007	MDCK2	Q136K	59.72 ± 3.83	53.3	0.13 ± 0.05	0.2	Resistant (S31N)
A/Myanmar/M114/2008	MDCK2	Q136K	33.37 ± 7.02	29.8	0.16 ± 0.03	0.2	Resistant (S31N)
A/Texas/131/2002§		None	1.43 ± 0.09	1.3	0.99 ± 0.09	1.2	Sensitive
A/Texas/131/2002_E119V§		E119V	5.43 ± 0.68	4.8	94.33 ± 2.06	109.7	Sensitive

*NA, neuraminidase;IC_50_, inhibitory concentration; NAI, neuraminidase inhibitors. †Amantadine sensitivity was based on M2 genotyping data. ‡Average IC_50_ was calculated excluding the control viruses (n = 47). §Reference strains used as drug-sensitive and -resistant control viruses in the NAI assay.

**Figure 1:**
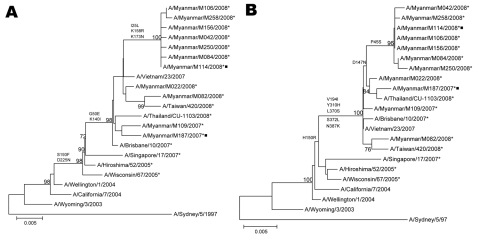
Phylogenetic analysis of the hemagglutinin (HA) (A) and neuraminidase (B) genes of influenza virus A (H3N2) isolates in Myanmar in 2007 and 2008. Trees were generated by using the neighbor-joining method. Bootstrap values >70% of 1,000 replicates and amino acid changes that characterize a branch are indicated on the left side of the node. Amantadine-resistant isolates with S31N mutation in M2 are marked with asterisks, and isolates with reduced sensitivity to zanamivir with Q136K mutation in NA are marked with squares. GenBank accession no. of the genomic sequences of isolates are GQ478849–GQ478866. Nucleotide sequences of the HA and NA genes of vaccine strains and isolates from other countries were obtained from the National Center for Biotechnology Information Influenza Virus Resource (www.ncbi.nlm.nih.gov/genomes/FLU). Scale bar indicates nucleotide substitutions per site.

**Figure 2:**
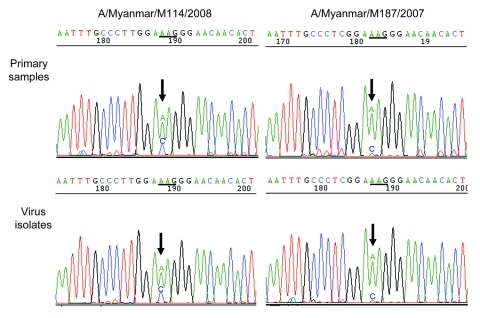
Detection of Q136K substitution in NA by sequencing in primary samples and virus isolates. Arrows indicate the first peak of the codon encoding amino acid position 136. Comparison of the sequence chromatogram showed a mixed population of bases in both original clinical samples and virus isolates, with a dominant peak for 136K (AAG) mutants, compared with wild-type 136Q (CAG) viruses. NA, neuraminidase.

Phylogenetic analysis of the HA and NA genes showed that the isolates with reduced sensitivity to zanamivir belonged to 2 distinct clusters (Figure 1). These viruses accumulated 2 and 3 amino acid (aa) substitutions in HA and 6 and 2 aa changes in NA in 2007 and 2008 (Figure 1), respectively. Epidemiologic and sequencing data did not suggest any link between the cases. Analysis of the NA gene showed that the isolates with reduced sensitivity to zanamivir had a glutamine (Q) to lysine (K) substitution at aa position 136. Sequence chromatograms showed a heterogeneous population of virus possessing either Q or K at position 136, with a dominant peak for the K136 mutant (Figure 2). Direct sequencing of primary samples showed a similar profile of chromatogram with a higher signal for the K136 mutant and a minor peak for the Q136 wild-type strain (Figure 2). The rest of the zanamivir-sensitive isolates in 2007 and 2008 had the Q136 genotype, and no NAI-resistant-associated mutations were detected elsewhere in the NA gene.

## Conclusions

In this study, we detected a novel influenza virus A (H3N2) with Q136K mutation in NA and S31N mutation in M2, which demonstrated reduced susceptibility to both zanamivir and amantadine but remained susceptible to oseltamivir. These Q136K viruses were isolated at a low frequency (<1.5%) in Myanmar in 2007 and 2008. Phylogenetic analysis showed that these viruses were already amantadine-resistant with S31N mutation in M2. Amantadine-resistant viruses with S31N mutation have been the predominant circulating strains among subtype H3N2 viruses in Myanmar since 2005 (*8*). The Q136K substitution in NA was probably generated by spontaneous point mutation. The HA and NA gene sequences of Q136K mutants were submitted to GenBank under accession nos. A/Myanmar/M187/2007: FJ229893 (HA), FJ229860 (NA) and A/Myanmar/M114/2008: GQ478854 (HA), GQ478863 (NA).

Hurt et al. recently reported the characterization of zanamivir-resistant subtype H1N1 with Q136K mutation in NA (*7*). Zanamivir IC_50_s of these viruses ranged from 6 nM to 238 nM (*7*), which differed from the 1–60 nM range of subtype H3N2 viruses obtained in this study. This finding may be due to differences in subtype and variations in the assay. The Q136K mutation was not detected in the primary clinical samples by sequencing (*7*), however, in our study, the Q136K mutation in subtype H3N2 isolates was detected in primary samples. Comparison of the sequence chromatograms between original samples and virus isolates showed a similar profile, suggesting that the Q136K mutants were present in primary samples of subtype H3N2 isolates. The presence of Q136K variants in primary samples appears to be subtype-specific because these mutants were present in very low proportions among subtype H1N1 viruses (*12*). To determine whether mutations exist in other gene segments associated with Q136K mutations, we performed a full genome analysis of Q136K mutants and wildtype viruses. We found no additional mutations in Q136K strains, which suggest that the genetic background of these viruses can compensate for the K136 mutation. However, further study is needed to confirm whether the accumulated 5 aa changes in HA and 8 substitutions in NA would compensate for the Q136K mutation.

We searched the database for NA sequences of influenza viruses A (H3N2) with Q136K mutation that are available on GenBank. Of the 3,381 sequences obtained, 4 sequences from human influenza, which were isolated in 1995, 2003, 2004, and 2007, and 1 sequence from swine influenza, which was isolated in Japan in 1997, contained the Q136K substitution. Sequences from Q136K mutants isolated before 2007 showed no mutations in the M2 gene. The data indicate that these viruses occur naturally because some of the isolates in the database were obtained before introduction of zanamivir into clinical practice in 1999 in Australia, New Zealand, United States, and Europe (*9*,*13*). In addition, Myanmar patients who shed these Q136K viruses did not receive any NAIs. The clinical relevance of Q136K mutants is unknown. Further study is needed to evaluate the effectiveness of zanamivir in patients infected with Q136K mutants.

Continued monitoring of viruses with reduced sensitivity to NAIs and adamantanes is needed, and routine surveillance should include both phenotypic and genotypic assays. The Q136K substitution in NA should be used as a molecular marker associated with reduced NAI susceptibility not only in subtype H1N1 isolates but also among subtype H3N2 isolates.
